# Primary central nervous system lymphoma: Inter‐compartmental progression

**DOI:** 10.1002/jha2.303

**Published:** 2022-01-20

**Authors:** Vishal Raval, Elaine Binkley, Mary E. Aronow, Juan Valenzuela, David M. Peereboom, Wei Wei, Sunil Srivastava, Jaqueline Davanzo, Herbert Culver Boldt, Mark P. McGarrey, George N. Papaliodis, Lucia Sobrin, Ivana K. Kim, Dimitrios G. Vavvas, Dean Eliott, Lakshmi Nayak, Emilio Dodds, Francisco Marco del Pont, Arun D. Singh

**Affiliations:** ^1^ Department of Ophthalmic Oncology Cole Eye Institute Cleveland Clinic Cleveland Ohio USA; ^2^ Department of Ophthalmology and Visual Sciences University of Iowa Iowa City Iowa USA; ^3^ Retina Service, Massachusetts Eye and Ear Harvard Medical School Boston Massachusetts USA; ^4^ Department of Retina and Ophthalmic Oncology Consultores Oftalmológicos Buenos Aires Argentina; ^5^ The Rose Ella Burkhardt Brain Tumor and Neuro‐Oncology Center Cleveland Clinic Cleveland Ohio USA; ^6^ Department of Quantitative Health Sciences Lerner Research Institute Cleveland Clinic Cleveland Ohio USA; ^7^ Center for Neuro‐Oncology Dana‐Farber Cancer Institute Boston Massachusetts USA; ^8^ Department of neurosurgery Fleni Buenos Aires Argentina

**Keywords:** inter‐compartment, primary central nervous system lymphoma, primary central nervous system lymphoma‐ocular variant, primary vitreoretinal lymphoma, progression, treatment

## Abstract

There is limited understanding of the inter‐compartmental progression and treatment outcomes of primary central nervous system lymphoma (PCNSL). In this multicenter retrospective cohort study on 234 patients with PCNSL (median age: 62.5 years [18–92]; median follow‐up 35 months [0.1–237.0]) from 2000 till 2018 were divided into group 1 (ocular, 44 patients): 1A and 1B without and with CNS progression and group 2 (CNS, 190 patients): 2A and 2B without and with ocular progression, respectively. In group 1 (44 patients), 33 patients received local treatment, and 11 patients received systemic treatment. In group 2 (15 patients), six patients received combination treatment, while seven patients received only systemic treatment. A complete response was observed in 19 (43%) and 91 (48%) patients in groups 1 and 2, respectively. The 2‐year progression‐free survival (PFS) was 35% (95% CI: 0.23, 0.54) and 56% (95% CI: 0.49, 0.63) for groups 1 and 2, respectively (*p* < 0.0001). Age < 60 years was significantly associated with longer PFS (median PFS 48 vs. 24 months, *p* = 0.01). The overall survival (OS) at 2‐year was similar among groups 1 and 2 (83% and 67%), respectively (*p* = 0.06). Thus, Initial compartment of involvement does not influence local response rate or OS.

## 1 INTRODUCTION

Primary central nervous system lymphoma (PCNSL) is a rare, aggressive, non‐Hodgkin lymphoma that is most frequently diffuse large B‐cell lymphoma (DLBCL) in origin [1]. It affects the central nervous system (CNS) compartments including the brain, cranial nerves, spinal cord, and meninges [1, 2]. PCNSL‐ocular (O), an ocular subset of PCNSL, is known to predominantly affect the ocular structures such as the sub‐retinal pigment epithelium or sub retinal space, retina, and vitreous. The ophthalmic manifestations can precede, occur simultaneously with, or follow extra‐ocular CNS disease. About 15%–25% of patients with PCNSL may present with or eventually develop ocular involvement [3]. Conversely, 56%–90% of patients with PCNSL‐O eventually involve the CNS compartment [1, 4]. An extensive review of studies on PCNSL indicates relatively infrequent recurrences outside the CNS compartments thereby demonstrating a unique tropism for the CNS [4]. As compared to DLBCL of sytemic origin, the overall prognosis is poor, and current treatment is comprised predominantly of methotrexate (MTX)‐based chemotherapy [5, 6, 7, 8].

In this study, *relapse* refers to recurrence of disease after a period of improvement within the initially affected compartment and *progression* refers to involvement of a previously unaffected compartment such as eye, the CNS, or vice versa. Currently, the management of PCNSL‐O is focused on local ocular control, given insufficient evidence that ocular treatment decreases progression to CNS. Despite achieving high rates of local ocular control with intravitreal agents including MTX [9] and rituximab [10], OS is poor as 65%–85% of patients eventually have disease progression into the CNS compartment followed by death within a median interval of 29 months [11, 12].

In order to eliminate tumor‐specific mortality due to CNS progression, outcome measures including progression‐free survival (PFS) and overall survival (OS) should be reported. We report inter‐compartmental progression and treatment outcomes including local control, PFS, and OS from a multicenter retrospective series of patients with PCNSL treated in the modern era and reflecting a real‐world scenario of patients treated by multidisciplinary teams [13].

## 2 METHODS

### 2.1 Study patients

Medical records of all patients diagnosed between January 1, 2000 and December 31, 2018 with PCNSL and PCNSL‐O (either preceding or following with CNS involvement) were reviewed and included in the study. Patients diagnosed with concurrent involvement of both the compartments were excluded from the final analysis. The study patients were pooled from four tertiary ocular oncology centers, three from the United States (Cleveland Clinic, Cleveland; University of Iowa, Iowa City; Harvard Medical School, Boston) and one from South America (Consultores Oftalmológicos, Buenos Aires, Argentina) (**Table S1**). All patients were aged ≥18 years, had pathological confirmation of DLBCL by vitreous or brain biopsy, and were immunocompetent with negative testing for human immunodeficiency virus. Staging evaluation in patients with biopsy confirmed diagnosis of PCNSL‐O was done by MRI brain with contrast along with lumbar puncture for cerebrospinal fluid (CSF) evaluation to rule out concurrent CNS involvement. This study was approved by the human research ethics committee of all the collaborating centers, and the study adhered to the tenets of the Declaration of Helsinki.

### 2.2 Study groups

Patients were divided into two groups based upon the compartment involved at initial presentation. Group 1: Patients with PCNSL‐O at presentation were sub‐grouped into 1A (limited to ocular compartment) and 1B (progressed into CNS compartment). Group 2: Patients with CNS only (PCNSL‐CNS) at presentation who were sub‐grouped into 2A (limited to CNS compartment) and 2B (progressed into ocular compartment).

### 2.3 Treatment regimens

Detailed treatment information and type of regimen were recorded for all patients. Patients who received ocular treatment were categorized by type of local ocular therapy: intravitreal therapy (MTX and/or rituximab) (**Table S2**), ocular radiation therapy, combination of intravitreal and radiation treatment, and others (pars plana vitrectomy or enucleation). CNS treatment included either local therapy (local excision, whole brain radiotherapy [WBRT], intrathecal MTX) or systemic therapy with intravenous high‐dose MTX (HD‐MTX) or rituximab, other chemotherapy combinations(procarbazine, vincristine, cytarabine, temozolamide, others), or use of myelo‐ablative chemotherapy with autologous stem cell transplant.

### 2.4 Outcome measures

The primary outcome measure was assessment of local treatment response in the eye and CNS compartments. Local treatment response was divided into complete response (CR), partial response (PR), progressive disease, or relapsed disease to mirror terminology and criteria proposed by International PCNSL collaborative group for standardization of baseline evaluation and response criteria for primary CNS lymphoma [14]. The secondary outcome measure was inter‐compartmental progression determined by PFS (defined as time from treatment initiation to disease progression into another compartment: ocular or CNS; brain parenchyma with or without CSF) or death, whichever occurred first. The tertiary outcome measure was OS defined as the time from initiation of treatment until death or last follow‐up. Ocular and systemic risk factors predictive of PFS and OS were also analyzed.

### 2.5 Statistical analysis

All continuous variables were reported as median, interquartile range, and range whereas categorical variables were reported as frequency and percentage. Fisher's exact test was used to compare patient characteristics between disease groups. Kaplan–Meier analysis was used to estimate PFS and OS at 2 and 5 years, and log‐rank tests were used to compare patient groups. Univariate and multivariate Cox proportional hazard models were performed to identify risk factors associated with PFS and OS. All tests were two‐sided, and *p*‐values < 0.05 were considered statistically significant. Statistical analysis was performed using SAS Studio 3.7 (SAS Institute, Cary, NC) and R version 3.6 (R Foundation, Vienna, Austria).

## 3 RESULTS

### 3.1 Patient characteristics

We identified 234 consecutive patients identified through ophthalmology practices only or through neuro‐oncology or both with newly diagnosed PCNSL/PCNSL‐O in our four‐center international ocular oncology consortium (four tertiary centers treated between January 1, 2000 and December 31, 2018 after excluding 15 patients with concurrent involvement of ocular and CNS compartment; eight patients with secondary CNS lymphoma and two patients with primary testicular lymphoma from 259 patients). The patient distribution in two groups was group 1 (PCNSL‐O, 44 patients), which were sub‐grouped into 1A (eight patients) and 1B (36 patients), and group 2 (PCNSL‐CNS, 190 patients), which were sub‐grouped into 2A (154 patients) and 2B (36 patients). The median time interval from symptom onset to diagnosis was 7 months and 2 months in groups 1 and 2, respectively (*p* < 0.0001). The median follow‐up of the entire cohort (234 patients) was 35 months (range 0.1–237.0 months) and was similar between groups (Table 1). Using reverse censoring, the median F/U was 73 months (95% CI: 60–87).

**TABLE 1:** Primary central nervous system lymphoma (PCNSL). Summary of patient characteristics by initial presentation

	Initial presentation						
	Group 1		Group 2		All		
	PCNSL‐O		PCNSL‐CNS				
Variable	N	%	N	%	N	%	*p*‐value
**Number**	44	100	190	100	234	100	
**Gender**							
Female	22	50	94	49	116	49	>0.99
Male	22	50	96	51	118	51	
**Ocular Involvement**							
OD	6	14	NA	NA	6	14	NA
OS	5	11	NA	NA	5	11	
OU	33	75	NA	NA	33	75	
**Ocular finding**							
Subretinal lesions +/− cells	23	52	14	7.37	37	46	0.27
Vitreous cells only	21	48	22	11.58	43	54	
**Ocular treatment**							
Local	33	75	25	13	58	72	0.0002
Systemic	11	25	11	6	22	28	
**Systemic treatment**							
HD MTX +/− chemo	26	72	118	62	144	67	0.0001
HD MTX +RT	5	14	67	35	72	33	
**Initial response (ocular)**							
Complete response	35	80	18	9	53	23	0.008
Refractory	9	21	18	9	27	12	
**Final response**							
Complete response	19	43	91	48	110	47	0.62
Relapse	25	57	99	52	124	53	
**Progression**							
No	8	18	154	81	162	65	N/A
Yes	36	82	36	19	72	29	
**Status**							
Alive	29	66	93	49	122	52	N/A
Dead	15	34	97	51	112	48	
**Follow‐up**							
Median	31.5	35.0	35.0	N/A			
Range	6.8–194.2	0.1–237.0	0.1–237.0				

*Note: p*‐values by Fisher's exact test.

Abbreviations: HD MTX, high dose methotrexate; OD, right eye; OS, left eye; OU, both eyes; PCNSL‐CNS, primary central nervous system lymphoma‐CNS only; PCNSL‐O, primary central nervous system lymphoma‐ocular only; RT, radiation therapy.

### 3.2 Primary outcome

#### 3.2.1 PCNSL‐O: Local control

In group 1 (44 patients), 33 patients received local treatment (intravitreal chemotherapy [*n* = 19], local radiation [*n* = 7], or both [n = 7]), and 11 patients received systemic chemotherapy for ocular control in absence of CNS disease. The initial ophthalmic CR was seen in 35 (80%) patients with nine (21%) patients being refractory to initial treatment received. At last follow‐up, the final ophthalmic CR was noted in 19 (43%) patients with ocular relapse occurring in 25 (57%) patients.

#### 3.2.2 PCNSL‐CNS: Local control

In our series of 190 patients with CNS disease at diagnosis, the median age at diagnosis was 62 years (range: 15–86 years). The median time interval from patients presenting with CNS symptoms to diagnosis of PCNSL was 2 months with median follow‐up of 35 months. A total of 118 (62%) received an HD‐MTX‐based regimens, while 62 (33%) received a combination of systemic chemotherapy and WBRT. At last follow‐up, relapse disease was recorded in 99 (52%) patients. In group 2B (36 patients), 24 patients had received HD‐MTX, and 12 patients received MTX‐based combination regimens as initial therapy.

### 3.3 Secondary outcome: 2‐year PFS

**FIGURE 1:**
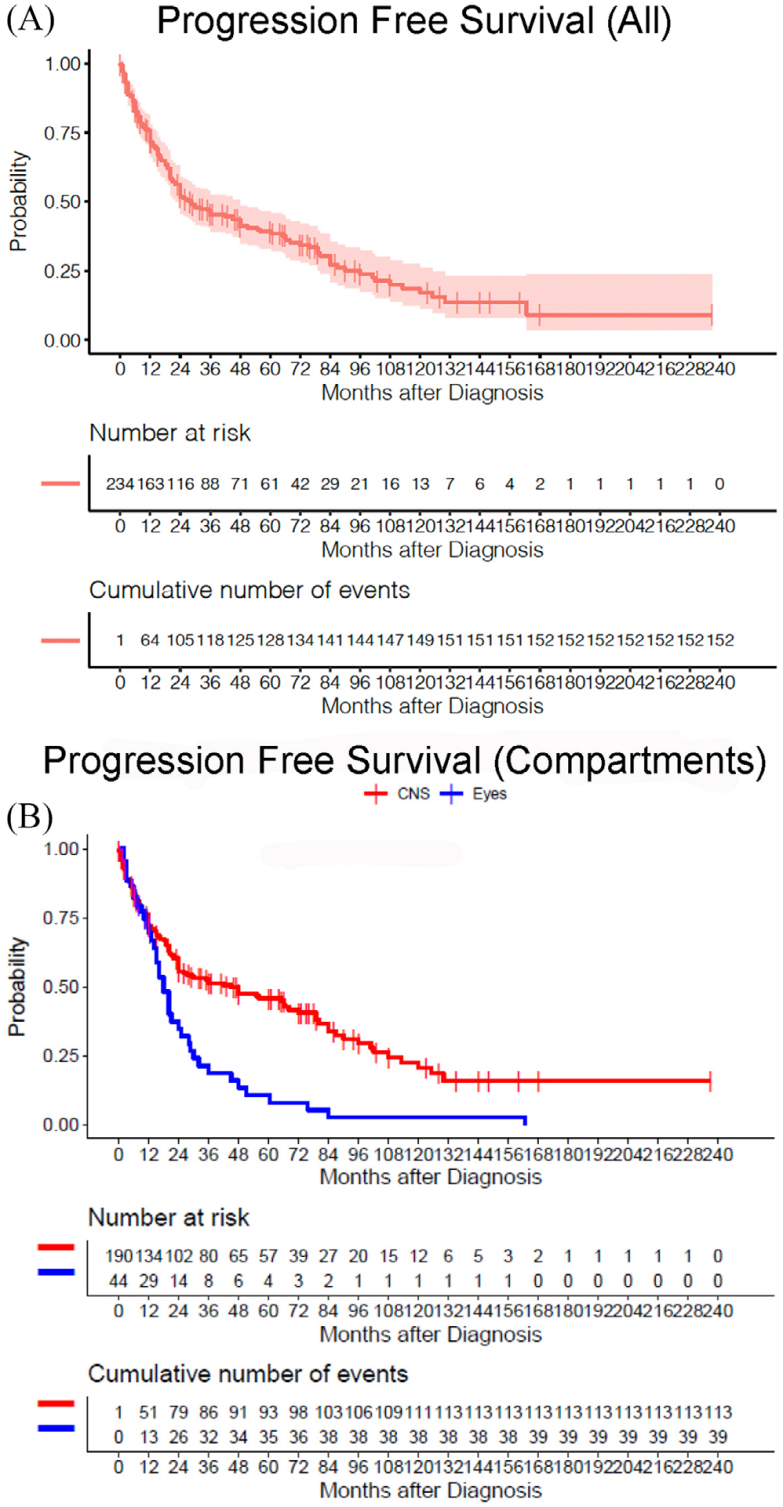
Progression‐free survival. Kaplan–Meier plot for entire cohort (A) and by groups defined by initial presentation (group 1 [PCNSL‐O] and group 2 [PCNSL‐CNS]) (B). Abbreviations: CNS, central nervous system; PCNSL, primary central nervous system lymphoma

Considering PCNSL‐O as an ocular subset of PCNSL, the intercompartmental progression of the disease from ocular to CNS compartments was noted in 36 (82%) patients (group 1B) and from CNS to ocular in 36 (19%) patients (group 2B). The median PFS of all patients was 28 months (95% CI: 22–48 months), and the 2‐year PFS rate was 52% (95% CI: 46%–59%) (Figure 1A). The median PFS depending upon initial presentation was 18 months (95% CI: 15–28 months) and 45 months (95% CI: 24–72 months) with 2‐year PFS of 35% (95% CI: 0.23, 0.54) and 56% (95% CI: 0.49, 0.63) for group 1 and group 2, respectively (*p* < 0.0001) (Figure 1B). Univariate regression analyses using a Cox models showed age < 60 years was significantly associated with longer PFS (*p* = 0.01), while sex, laterality, specific clinical features such as vitreous cells or subretinal infiltration, and type of ocular therapy were not significant (*p* > 0.05) (Table S3).

Within group 1, the impact of ocular therapy on PFS was not statistically significant. The median PFS was 18 (95% CI: 14–24) and 16 (95% CI: 6‐NA) months, for individuals treated with local ocular therapy versus systemic therapy, respectively. The 2‐year PFS rate was 36% (95% CI: 23–58 months) and 29% (95% CI: 9–90) for those treated with local ocular therapy and systemic therapy, respectively (*p* = 0.8).

### 3.4 Tertiary outcome: 2‐year overall survival

**FIGURE 2:**
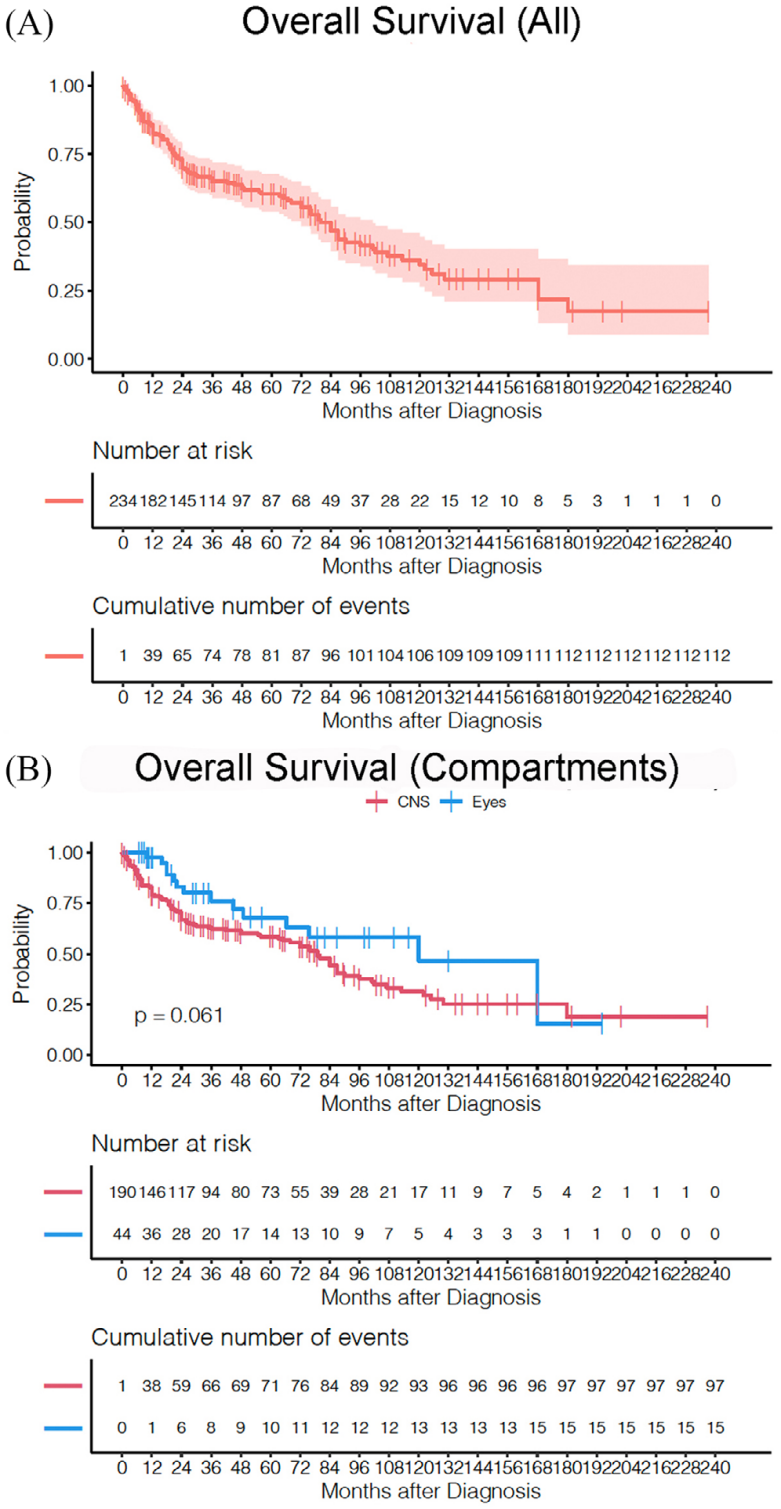
Overall survival. Kaplan–Meier plot for entire cohort (A) and by groups defined by initial presentation (group 1 [PCNSL‐O] and group 2 [PCNSL‐CNS])(B). Abbreviations: CNS, central nervous system; PCNSL, primary central nervous system lymphoma

**FIGURE 3:**
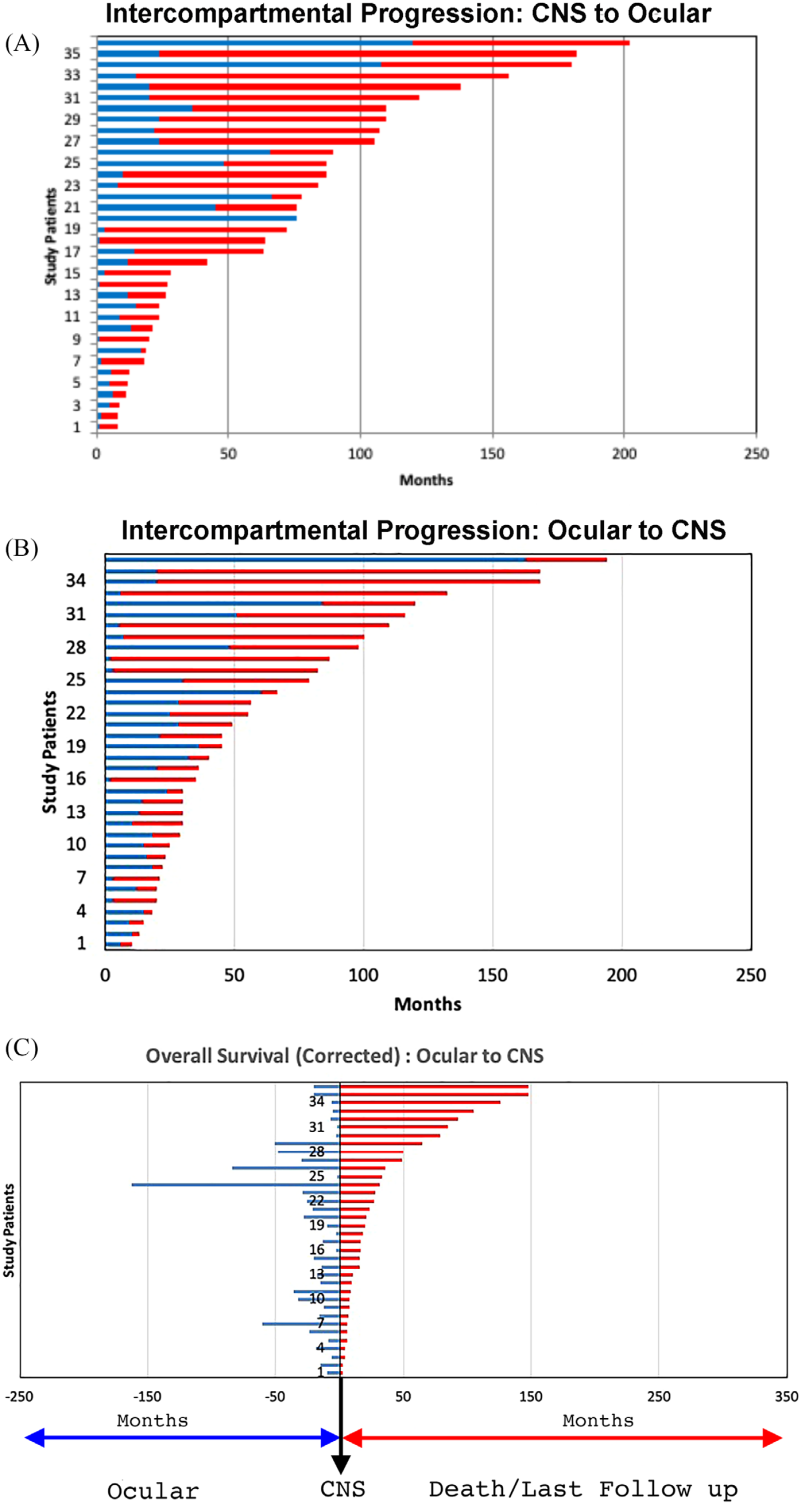
Swimmer plot depicting intercompartmental progression of individual patients. The overall survival (OS) of patients in group 2B (central nervous system [CNS] to ocular)(A) and group 1B (ocular to CNS)(B) is divided into two periods. The blue line denotes the intercompartmental progression (progression‐free survival [PFS]), and red line denotes the remaining duration of survival/last follow‐up. The corrected OS (duration of patient survival from the time of CNS diagnosis) in group 1B patients (C). The blue line denotes time until CNS progression (PFS), and red line is the corrected survival. PFS in this group of patients induces lead time bias

The median OS for the entire cohort was 80 months (95% CI: 72–102 months) with 2‐ and 5‐year OS rates of 70% (95% CI: 64%–76%) and 60% (95% CI: 54%–68%), respectively (Figure 2A). Depending upon the initial presentation, the median OS was 120 (95% CI: 66.5‐NA) and 79 (95% CI: 65–90) months with 2‐year OS of 83% and 67% for groups 1 and 2, respectively (*p* = 0.06) (Figure 2B). Univariate regression analyses using a Cox model log‐rank test showed that none of the factors such as age, initial presentation, sex, laterality, ocular findings such as vitreous cells or subretinal infiltration (in any one eye) or treatment regimen used were found to be statistically significant (*p* > 0.05) (Table S4). When corrected for lead time bias (PFS, median 18 months, 95% CI: 15–28 months) in group 1B, the median OS was reduced to 102 months (95% CI: 31–93) (Figure 3).

**FIGURE 4:**
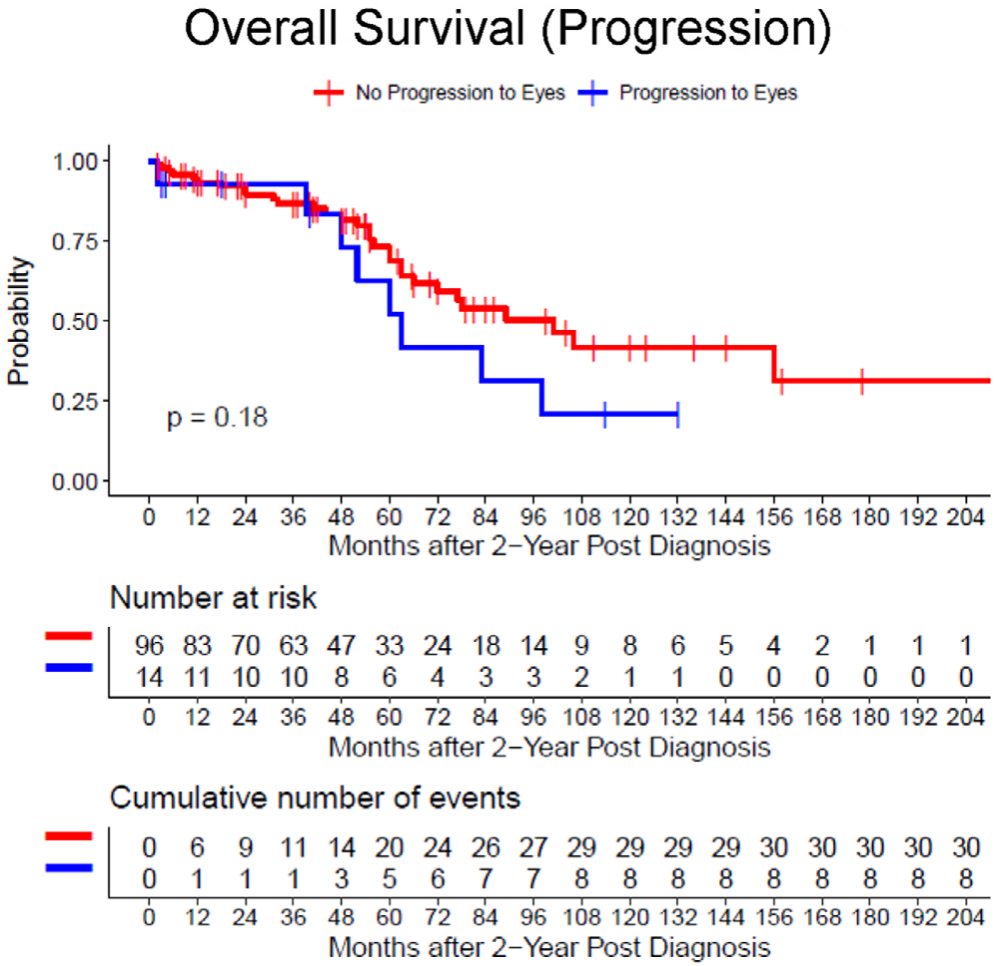
Overall survival in group 2 (primary central nervous system lymphoma [PCNSL]‐central nervous system [CNS]). Kaplan–Meier plot for patients without (group 2A) and with (group 2B) ocular progression

In addition, there was no significant difference in OS between patients with disease limited to the CNS compartment (group 2A) versus those that progressed to the ocular compartment (group 2B) (101 vs. 63 months, *p* = 0.18) based on landmark analysis at 2‐year post‐initial diagnosis (Figure 4).

## 4 DISCUSSION

Currently, the treatment goal in the management of PCNSL‐O is focused on local control, given limited evidence that systemic therapy reduces CNS progression [12]. Despite achieving high rates of local ocular control with intravitreal agents like MTX [9] and rituximab [10], OS is poor as 65%–85% of patients ultimately progress to involve the CNS followed by death within a median interval of 29 months [11]. We have reported a well‐defined and informative set of outcome measures that can be used in future studies. Patients were treated by multidisciplinary teams, and we were unable to discern subtle differences in practice patterns between centers.

### 4.1 Local response

The initial ophthalmic CR rate of 80% and 50%, in group 1 and 2B patients, respectively was significantly different between the groups (*p* = 0.008). In the largest published series of patients (122 eyes of 74 patients) treated with intravitreal MTX, local control was achieved in all cases (100%) following two to 16 intravitreal injections [15]. Although treated at different institutions, the intravitreal MTX protocol was similar between institutions comprising induction, consolidation, and maintenance phases (Table S2).

The role of HD‐MTX‐based systemic chemotherapy used as first‐line treatment in management of PCNSL‐O remains controversial. A small study by Batchelor et al.analyzed ocular response to systemic HD‐MTX, where seven of nine patients showed good initial response (six CRs and one PR but two patients had persistent disease despite achieving therapeutic micromolar concentrations of systemic HD‐MTX) [7]. Given the relatively high rate of neurotoxicity, risk of infection, and other side effects from systemic chemotherapy, it is not widely accepted as the primary method of treatment in patients with PCNSL‐O. Presently, the role of systemic chemotherapy with/without local treatment is generally limited to individuals with refractory/relapsed PCNSL‐O or patients who have concurrent CNS involvement.

### 4.2 Other outcome measures

Given that PCNSL‐O is a subset of PCNSL [16] with a strong propensity to progress to the CNS (intercompartmental progression), treatment effectiveness should not be evaluated soley in terms of local ocular response [12]. Instead, evaluation of outcomes such as PFS and OS are critical. In this study, relapse refers to recurrence of disease after a period of improvement within the previously affected compartment, and progression refers to involvement of the previously unaffected compartment such as ocular to CNS involvement or vice versa. Based upon the natural history of PCNSL‐O, intercompartmental progression is an important event for capturing of PFS, a critical outcome measure to assess overall the impact of ocular therapy. An intervention that prolongs PFS can be expected to improve OS, as death from PCNSL‐O is due to CNS progression. Therefore calculation of OS in patients with only ocular disease at presentation (group 1) can be misleading unless time to CNS progression (PFS) is also reported to adjust for lead time bias (Figure 3). The interest in PFS also stems in part from the fact that some treatment strategies are aimed only toward stabilization of the disease thereby reducing the morbidity. Trials that report PFS may be conducted more quickly using fewer subjects and at lower costs than those incorporating OS [17]. It is important to emphasize that mere improvement of PFS does not always translate to an increase in OS, hence the great importance of analyzing OS outcomes.

Most retrospective studies (Table S5) have defined PFS as time from onset of diagnosis to progression or relapse/death [18, 19, 20, 21, 22]. PFS defined in this way results in heterogenous outcomes such as local relapse, progression, and death. Therefore, by including relapse as an event for PFS leads to overestimation of OS. The lack of well‐defined outcome measures, particularly related to intercompartmental progression (PFS), further hampers valid comparisons between published studies. To overcome these limitations, we have purposefully calculated PFS as time to intercompartmental progression adding to the efforts to standardize reponse criteria as it relates to PCNSL‐O [14]. Our results showing a median PFS of 18 months (95% CI: 15–28 months) for group 1B patients is comparable to the Korean consortium for improving survival of lymphoma (CISL) study [23], which defined PFS as compartmental progression of CNS and found a median PFS of 25.4 months (95% CI, 18–48.3 months).

### 4.3 Impact of PCNSL‐O on PFS and OS

A few studies have demonstrated a lack of prognostic impact of ocular involvement [18, 20, 24], while others have shown that ocular involvement at diagnosis has a negative prognostic impact on PFS and OS [25, 26]. The discrepancy in findings can be explained by the fact that these studies were retrospective and included heterogenous treatment regimens with HD‐MTX‐based regimens in only 55%–84% of patients, in contrast to studies showing poorer PFS and OS in the ocular involvement group, which were prospective and included HD‐MTX‐based chemotherapy regimens for all patients. In our series, we also show that there is no prognostic impact of ocular involvement (preceding, or following CNS involvement) on median OS when compared among the three groups (*p* = 0.1). Regression analyses showed that patients with older age (≥60 years) and the type of ocular involvement (vitreous seeding vs. sub‐RPE infiltrates) were not a significant factor, similar to previous observations [27].

### 4.4 Impact of route of therapy for PCNSL‐O on PFS and OS

Given the propensity for PCNSL‐O to progress into the CNS, an important question remains as to whether initial treatment of PCNSL‐O should be with local ocular therapy, systemic therapy, or a combined approach. Several single‐institution studies and multicenter retrospective studies including a report from the international PCNSL collaborative group (IPCG) have shown no difference in CNS progression or 5‐year OS when comparing patients treated with local, systemic, and or combined therapy [19, 20, 21, 27]. In contrast, a large multicenter retrospective study by Castellino et al. [28] and a single‐center study by Hashida et al. [29] have shown that PCNSL‐O patients receiving combined treatment had improved PFS as compared to those who received local treatment alone. Similarly, in a prospective study, Akiyama et al. [23] showed that for a group receiving combined treatment (*n* = 18) compared to a group with local treatment (*n* = 8), 2‐year PFS rates were 58.3% (95% CI: 23.0%–82.1%) and 37.5% (95% CI: 8.7%–67.4%), respectively. In addition, a study by Hormigo et al. [25] showed that patients who received systemic chemotherapy for local ocular disease before CNS progression had a significantly improved OS when compared to patients who received systemic treatment after CNS progression (39 months vs. 24 months, *p* < 0.03). Due to small number of cases in each study, sub‐groups (≤20), and heterogeneous treatment regimens, all of the published studies to date appear to be underpowered to be able to demonstrate a statistically significant impact on PFS between those treated with local ocular versus systemic therapy.

### 4.5 Limitations

The major limitation in all large, retrospective, multicenter studies of PCNSL are the length of study duration and the possibility that treatment regimens may change over time. Because of retrospective nature of this study, data on toxicity and neurotoxicity are lacking in this study. Another limitation is case selection bias depending on whether PCNSL‐O or PCNSL disease is the presenting manifestation. Our study was over the past 18 years, a period during which treatments have consistently relied primarily on HD‐MTX‐based regimens. Lack of sufficient sample size in the sub‐groups of patients treated with different treatment regimens.

### 4.6 Conclusions

Initial compartment involvement does not appear to influence the local response rate and OS (2 years). However, variability of PFS between groups reflects the natural history of intercompartmental progression of PCNSL. Ocular involvement (prior, or subsequent to CNS involvement) had no impact on OS in the presence of CNS disease. Although intravitreal MTX provides excellent local ocular control, impact on CNS progression cannot be expected from local treatment. Systemic therapy has theoretical possibility of reducing or eliminating CNS progression. However, current literature does not conclusively support superiority or inferiority of ocular therapy compared to systemic therapy for treatment for PCNSL‐O [30]. To answer such specific questions of great relevance, a large consortium of participating institutions is needed to generate prospective datasets to provide the statistical power.

## Supporting information

Supporting Information

Supporting Information

Supporting Information

Supporting Information

Supporting Information
