# Supplementary material for: Primary central nervous system lymphoma: Inter‐compartmental progression
Source: EJHaem. 2022 Jan 20;3(2):362–70. doi: 10.1002/jha2.303 (PMC9175875; doi:10.1002/jha2.303)
Supplement: Supplementary file 1 — Supporting Information [file JHA2-3-362-s004.docx]

**Supplement Table. 1 Sources of pooled multi institutional data. Involvement at presentation. Analysis of combined cases not included in the present study.**

| Institution | Ocular (n=44) | CNS (n=190) | Combined (n=15) |
| --- | --- | --- | --- |
| CEI, Cleveland | 19 | 95 | 04 |
| MEEI, Boston | 08 | 28 | 04 |
| UOI, Iowa City | 11 | 47 | 04 |
| CO, Buenos Aires | 06 | 20 | 03 |
