# Supplementary material for: Primary central nervous system lymphoma: Inter‐compartmental progression
Source: EJHaem. 2022 Jan 20;3(2):362–70. doi: 10.1002/jha2.303 (PMC9175875; doi:10.1002/jha2.303)
Supplement: Supplementary file 2 — Supporting Information [file JHA2-3-362-s001.docx]

**Supplement Table 2. Treatment protocol for intravitreal methotrexate injection (400 microg/0.1 ml) included induction, consolidation, and maintenance phases based on Frenkel S, Hendler K, Siegal T, Shalom E, Pe'er J. Intravitreal methotrexate for treating vitreoretinal lymphoma: 10 years of experience. Br J Ophthalmol. 2008 Mar;92(3):383-8.**

| Institution | Induction | Consolidation | Maintenance |
| --- | --- | --- | --- |
| CEI, Cleveland | Twice weekly: 4 weeks | Weekly: 8 weeks | Monthly: 9 months |
| MEEI, Boston | Twice weekly: 4 weeks | Weekly: 8 weeks | Monthly: 9 months |
| UOI, Iowa City | Twice weekly: 4 weeks | Weekly: 4 weeks  Every 2 weeks: 8 weeks | Monthly: 8 months |
| CO, Buenos Aires | Twice weekly: 4 weeks | Weekly: 4 weeks | Monthly: 10 months |
