# Supplementary material for: Primary central nervous system lymphoma: Inter‐compartmental progression
Source: EJHaem. 2022 Jan 20;3(2):362–70. doi: 10.1002/jha2.303 (PMC9175875; doi:10.1002/jha2.303)
Supplement: Supplementary file 3 — Supporting Information [file JHA2-3-362-s002.docx]

**Supplement Table 3. PCNSL. Determinants of progression-free survival by patient group.**

| Factor | Level | Total N | Number of Events | Median Survival  (months?) (95%CI) | Rate at 2 Years (95%CI) | P-value |
| --- | --- | --- | --- | --- | --- | --- |
| **All patients** |  | 234 | 152 | 28 ( 22 , 48 ) | 0.52 ( 0.46 , 0.59 ) |  |
| **Initial**  **Compartment** | Ocular | 44 | 39 | 18 ( 15 , 28 ) | 0.35 ( 0.23 , 0.54 ) | < 0.0001 |
|  | CNS | 190 | 113 | 45 ( 24 , 72 ) | 0.56 ( 0.49 , 0.63 ) |  |
| **Age (years)** | <60 | 97 | 57 | 48 ( 24 , 84 ) | 0.58 ( 0.48 , 0.69 ) | 0.01 |
|  | ≥60 | 137 | 95 | 24 ( 19 , 36 ) | 0.48 ( 0.4 , 0.57 ) |  |
| **Gender** | Female | 116 | 79 | 24 ( 20 , 48 ) | 0.48 ( 0.39 , 0.58 ) | 0.1 |
|  | Male | 118 | 73 | 32 ( 24 , 75.6 ) | 0.56 ( 0.47 , 0.66 ) |  |
| **Ocular involvement** | OD | 12 | 11 | 20 ( 15 , NA ) | 0.19 ( 0.05 , 0.65 ) |  |
|  | OS | 14 | 14 | 9.5 ( 6 , 36 ) | 0.14 ( 0.04 , 0.52 ) | 0.1 |
|  | OU | 54 | 50 | 16.7 ( 13 , 24 ) | 0.33 ( 0.22 , 0.49 ) |  |
| **Ocular Finding** | Subretinal lesions +/- cells | 37 | 36 | 13 ( 8.5 , 17.9 ) | 0.23 ( 0.12 , 0.42 ) | 0.3 |
|  | Vitreous cell only | 43 | 39 | 20 ( 15 , 30 ) | 0.32 ( 0.2 , 0.51 ) |  |
| **Ocular Treatment** | Local | 33 | 31 | 18 ( 14 , 28.5 ) | 0.36 ( 0.23 , 0.58 ) | 0.8 |
|  | Systemic | 11 | 8 | 16 ( 15 , NA ) | 0.29 ( 0.09 , 0.9 ) |  |

P-values by log rank test
