# Supplementary material for: Primary central nervous system lymphoma: Inter‐compartmental progression
Source: EJHaem. 2022 Jan 20;3(2):362–70. doi: 10.1002/jha2.303 (PMC9175875; doi:10.1002/jha2.303)
Supplement: Supplementary file 4 — Supporting Information [file JHA2-3-362-s003.docx]

**Supplement Table 4. PCNSL. Determinants of overall survival (OS) by patient group.**

| Factor | Level | Total N | Number of Events | Median Survival (months) (95%CI) | Rate at 2 Years (95%CI) | Rate at 5 Years (95%CI) | P-value |
| --- | --- | --- | --- | --- | --- | --- | --- |
| All patients |  | 234 | 112 | 80 ( 72 , 101 ) | 0.7 ( 0.64 , 0.76 ) | 0.6 ( 0.54 , 0.68 ) |  |
| **Initial compartment** | CNS | 190 | 97 | 79 ( 65 , 90 ) | 0.67 ( 0.6 , 0.74 ) | 0.58 ( 0.51 , 0.66 ) |  |
|  | Ocular | 44 | 15 | 120 ( 66.5 , NA ) | 0.83 ( 0.71 , 0.96 ) | 0.68 ( 0.53 , 0.87 ) |  |
| **Age** | <60 | 97 | 42 | 96 ( 84 , NA ) | 0.74 ( 0.66 , 0.84 ) | 0.67 ( 0.57 , 0.78 ) | 0.06 |
|  | ≥60 | 137 | 70 | 72 ( 48 , 90 ) | 0.67 ( 0.59 , 0.76 ) | 0.56 ( 0.47 , 0.66 ) |  |
| **Gender** | Female | 116 | 56 | 84 ( 72 , 113 ) | 0.69 ( 0.6 , 0.78 ) | 0.64 ( 0.55 , 0.74 ) | 0.7 |
|  | Male | 118 | 56 | 76 ( 55 , 125 ) | 0.71 ( 0.63 , 0.8 ) | 0.57 ( 0.48 , 0.68 ) |  |
| **Ocular**  **Finding** | Subretinal lesions +/- cells | 37 | 16 | NA ( 63.1 , NA ) | 0.72 ( 0.59 , 0.88 ) | 0.65 ( 0.51 , 0.83 ) | 0.9 |
|  | Vitreous cells only | 43 | 26 | 107 ( 75.6 , NA ) | 0.83 ( 0.73 , 0.95 ) | 0.68 ( 0.56 , 0.84 ) |  |
| **Ocular Treatment**  **(Group 1)** | Local | 33 | 10 | 120 ( 66.5 , NA ) | 0.86 ( 0.74 , 1 ) | 0.71 ( 0.54 , 0.92 ) | 0.3 |
|  | Systemic | 11 | 5 | 75.6 ( 21 , NA ) | 0.69 ( 0.4 , 1 ) | 0.51 ( 0.14 , 1 ) |  |
| **Systemic Treatment**  **(Group 2b)** | HD MTX +/- chemo | 24 | 13 | 84 ( 72 , NA ) | 0.74 ( 0.58 , 0.94 ) | 0.74 ( 0.58 , 0.94 ) | 0.9 |
|  | HD MTX +RT | 12 | 7 | 87 ( 26 , NA ) | 0.74 ( 0.53 , 1 ) | 0.65 ( 0.42 , 1 ) |  |

P-values by log rank test

NA: Not available because 50% survival not achieved at the end of the available data. Even if median survival has been reached in a group, it was not be possible to calculate complete confidence intervals for those median values.
