# Supplementary material for: Primary central nervous system lymphoma: Inter‐compartmental progression
Source: EJHaem. 2022 Jan 20;3(2):362–70. doi: 10.1002/jha2.303 (PMC9175875; doi:10.1002/jha2.303)
Supplement: Supplementary file 5 — Supporting Information [file JHA2-3-362-s005.docx]

**Supplement Table 5. Published studies reporting survival outcomes (PFS and OS) in PCNSL.**

| **Author (Year)** | **N** | **Inter-**  **compartmental Progression (%)** | **Median time to progression**  **(months)** | **PFS**  **rate** | **Median OS (months)** | **OS rate** | **PFS Definition** |
| --- | --- | --- | --- | --- | --- | --- | --- |
| **PCNSL-O patients** | | | | | | | |
| **Riemens (2015)** | 78 | 28 (36) | 29 | NA | 44 | 35% (5-year) | From symptoms to intercompartmental progression, death or last  follow-up |
| **Castellino (2019)** | 33 | 10 (30) | 30 | NA | 111 | NA | From diagnosis to relapse or progression in any compartment |
| **Cho**  **(2018)** | 14 | 11 (79) | 17.4 | NA | 37 | NA | Not defined |
| **Lee**  **(2015)** | 6 | 4 (67) | 25.4 | NA | NA | 90% (3-year) | From diagnosis to intercompartmental progression, death or last  follow-up |
| **Grimm (2007)** | 83 | 29 (35) | 19 | NA | 58 | NA | From diagnosis to relapse, progression or death |
| **Dalvin (2020)** | 27 | 14 (52) | 35 | NA | 49 | NA | Not defined |
| **Present**  **study**  **(2021)** | 44 | 36 (82) | 18 | 35% (2-year) | 120 | 68% (5-year) | From treatment to progression into other compartment or death |
| **PCNSL-CNS patients** | | | | | | | |
| **Zhuang (2019)** | 103 | 21 (20) | 13 | 24% | 51 | 62% (2-year) | Time from entry into the study to the first sign of progression |
| **Cho (2018)** | 18 | 18 (100) | 32.4 | NA | 44 | NA | Not defined |
| **Lee**  **(2015)** | 9 | 9 (100) | 12.5 | NA | NA | 53% (3-year) | From diagnosis to progression, into other compartment, death, or the last follow-up |
| **Dalvin (2020)** | 50 | 36 (72) | 16 | NA | 48 | NA | Not defined |
| **Houillier**  **(2021)** | 1002 | 1002 (100) | 8 | 36% (2- year) | 25.3 | 38% (5- year) | Time between the diagnosis and the progression of the disease or death |
| **Present**  **study**  **(2021)** | 190 | 36 (19) | 45 | 56% | 79 | 58% (5-year) | From treatment to progression into other compartment or death |

PCNS: Primary central nervous system lymphoma

PCNSL-O: Primary central nervous system lymphoma-ocular only

PCNSL-CNS: Primary central nervous system lymphoma- CNS only
